# Editorial: Novel pharmacological targets and strategies to treat neglected global diseases (NGDs): an LMIC perspective

**DOI:** 10.3389/fphar.2024.1527705

**Published:** 2025-01-06

**Authors:** Kishor M. Wasan, Somia Iqtadar, Celestin Nzanzu Mudogo, Miguel Angel Chávez-Fumagalli

**Affiliations:** ^1^ The Neglected Global Diseases Initiative, The University of British Columbia, Vancouver, BC, Canada; ^2^ Department of Urologic Sciences, Faculty of Medicine, The University of British Columbia, Vancouver, BC, Canada; ^3^ Department of Medicine, King Edward Medical University, Lahore, Pakistan; ^4^ Unit of Molecular Biology, Department of Basic Sciences, Faculty of Medicine, University of Kinshasa, Kinshasa, Democratic Republic of the Congo; ^5^ Computational Biology and Chemistry Research Group, Vicerrectorado de Investigación, Universidad Católica de Santa María, Arequipa, Peru

**Keywords:** pharmacology, neglected diseases, COVID, malaria, leishmaina

## Summary of research topic

Neglected Global Diseases (NGDs) are infectious diseases and other conditions that cause physical and cognitive impairments, contribute to mother and child illness and death, and make it difficult to earn a living, thereby disproportionately affecting the world’s poorest populations. They include maternal, infant, and child health conditions, neglected tropical diseases (NTDs), as well as HIV/AIDS, tuberculosis, and malaria. The World Health Organization (WHO) has a list of 21 NTDs that cause substantial illness for more than one billion people globally (World Health Organization, 2024). These diseases thrive in conditions of extreme poverty, areas that generally have unsafe water, poor sanitation, substandard housing, and limited access to healthcare or essential medicines. In some countries, this includes most rural areas, urban slums, or conflict zones. However, it is increasingly recognized that emerging middle-income countries contain the highest numbers of people affected.

NGDs impact daily life for many, predominantly in countries with weak health and social support systems, they cripple families and contribute to the harsh cycle of poverty. While some NTDs are fatal without treatment, most of them are debilitating and stigmatizing. Children are particularly vulnerable. Overall, NGDs cause great suffering and are a leading cause of chronic disability, diminished quality of life, and premature death. Despite their impact, funding for NTDs research and treatment fades compared to “developed world impacted or supported diseases.”

Unfortunately, particularly in the past 20 years, the development of novel pharmacological targets and new interventions based on these targets to treat NGDs has been limited (Ferreira et al., 2022). However, in recent years there has been a resurgence of research in these areas from low- or middle-income countries (LMICs) researchers themselves. Unlike similar Research Topics in this area, the purpose of this Research Topic was to highlight the novel work of LMIC researchers in developing new targets and interventions to treat NGDs.

## Contributing articles

We received five contributions from colleagues highlighting everything from Global Access Principles to research on leishmaniasis.

Gallini et al. discussed the progress of the University of British Columbia over the past 15 years on several technologies that fall under their Global Access and Equity principles. The technologies reported on are wide-ranging, including an oral medication for the treatment of leishmaniasis; peptides for potential use against malaria and various bacterial, viral, and fungal infections; a portable vaccine cooler; a diagnostic technology to detect severe sepsis; and an SMS Messaging System to monitor and support patients with HIV, TB and COVID-19. The article identified challenges that the researchers face in implementing these technologies’ principles and potential solutions for overcoming them through creative licensing and partnerships with public and private sectors, governments, local companies, and communities.

Bess et al., in their article entitled “*Identification of oral therapeutics using an AI platform against the virus responsible for COVID-19, SARS-CoV-2*,” discussed a sophisticated computational pipeline, eVir, designed for the discovery of antiviral drugs based on their interactions within the human protein network. The researchers devised an Artificial Intelligence (AI) system to explore repurposing opportunities for currently used oral therapies. The eVir system operates by identifying pharmaceutical compounds that mirror the effects of antiviral peptides (AVPs)—fragments of human proteins known to interfere with fundamental phases of the viral life cycle: entry, fusion, and replication. eVir extrapolates the probable antiviral efficacy of a given compound by analyzing its established and predicted impacts on the human protein-protein interaction network. This innovative approach provides a promising platform for drug repurposing against SARS-CoV-2 or any virus for which peptide data is available.

Goyzueta Mamani et al., provided research from their paper entitled “*Targeting Leishmania infantum Mannosyl-oligosaccharide glucosidase (MOGS) with natural products: potential pH-dependent inhibition explored through computer-aided drug design*” where they screened natural products (NP) from Nuclei of Bioassays, Ecophysiology, and Biosynthesis of Natural Products Database (NuBBEDB), Mexican Compound Database of Natural Products (BIOFACQUIM), and Peruvian Natural Products Database (PeruNPDB) databases, in addition to structural analogs of Miglitol and Acarbose, which have been suggested as treatments for VL and have shown encouraging action against parasite’s N-glycan biosynthesis. Their findings suggested that Ocotillone and Subsessiline have potential antileishmanial effects at pH 5 and 7, respectively, due to their high binding affinity to MOGS and interactions in the active center. Furthermore, these compounds were non-toxic and had the potential to be administered orally. This research indicates the promising anti-leishmanial activity of Ocotillone and Subsessiline, suggesting further validation through in vitro and in vivo experiments.

Lokole et al., in their review paper entitled “*Plant-based nanoparticles targeting malaria management*,” summarized studies on the use of plant-derived nanoparticles as cost-effective preventative measures against malaria parasites, starting from the vector stage. They also reviewed plant-based nanoengineering strategies to target malaria parasites and further discussed the site-specific delivery of natural products using ligand-decorated nanoparticles that act through receptors on the host cells or malaria parasites. The investigation of traditionally established plant medicines, surface-engineered nanoparticles, and the molecular targets of parasite and host cells may yield significant insights for the future development of antimalarial drugs. This research could potentially open new pathways for scientific advancement aimed at the eradication of malaria.

Quadros-Gomes et al., in their paper entitled “*Impact on parasitemia, survival time and pro-inflammatory immune response in mice infected with Plasmodium berghei treated with Eleutherine plicata*,” reported on pro-inflammatory changes in mice infected with Plasmodium berghei and correlated these changes with parasitemia and survival. EEEp, FDMEp, and eleutherol showed activity on the 5th day of infection, with only FDMEp being active on the 8th day. Treatment with EEEp and FDMEp extended animal survival, reduced IFN-γ and NO levels, and increased IL-10 levels. Eleutherol significantly altered the response, with eleutherol glucuronide seemingly active by binding to lactate dehydrogenase, inhibiting hemozoin metabolism and leading to parasite death. Pro-inflammatory changes did not appear to correlate with survival and reduced parasitemia. In summary, FDMEp and eleutherol reduced parasitemia, extended survival, and modulated the inflammatory response. FDMEp and eleutherol are promising candidates for developing new antimalarial drugs.

Taken together these five articles provide preliminary insight into the breadth and depth of research activities being conducted around the world to address NTDs. The future is bright as these research programs continue to flourish.
